# Equal Access, Equal Outcomes: Telehealth Utilization Around the COVID-19 Pandemic among People Living with HIV and Opioid Use Disorder in the Deep South

**DOI:** 10.1007/s10461-024-04550-5

**Published:** 2024-11-15

**Authors:** William S Bradford, Julie England, Reed W R Bratches, Ellen F Eaton

**Affiliations:** 1 Division of Infectious Diseases, University of Alabama at Birmingham, Boshell Building 8th floor 1808 7th Ave S, Birmingham, AL 35233, USA; 2 Department of Medicine, University of Alabama at Birmingham, Birmingham, USA; 3 School of Nursing, University of Alabama Birmingham, Birmingham, USA

**Keywords:** Telehealth, HIV, Deep South, COVID-19

## Abstract

Telehealth has the potential to extend access to lifesaving treatment for opioid use disorder (OUD) among underserved people living with HIV (PWH). However, policymakers have scaled back pandemic-era telehealth provisions, citing concerns about safety and effectiveness. In this study of 42 PWH with OUD in one Deep South HIV clinic between 3/1/2020 and 4/30/2021, we used multivariable regression to assess the impact of telehealth utilization on patient-centered HIV and OUD outcomes. We found no significant difference in outcomes for those with high telehealth utilization versus others. In addition to being more accessible, telehealth does not appear to compromise health outcomes.

## Background

Opioid use disorder (OUD) in rural areas is a major driver of the HIV epidemic, but access to care for addiction in these areas is generally poor. Alabama is one of 7 priority states in the *Ending the HIV Epidemic (EHE)* initiative due to a large rural burden of HIV. Yet, the state suffers from particularly limited access to substance use care with a shortage of providers, lack of Medicaid expansion, and state-imposed limitations on syringe service programs and other harm reduction measures [1, 2]. Telehealth delivery offers improved OUD treatment access for rural patients but, in some states, is limited by stringent government regulations, uncertainty around safety and efficacy, and payer restrictions [3–5]. The COVID-19 pandemic provided a unique opportunity for ecological study of telehealth for OUD treatment as governments and payers relaxed longstanding restrictions on virtual care to respond to the public health emergency [3, 6]. Data from this period are still emerging but suggest that increased telehealth use was effective in promoting medications for opioid use disorder (MOUD) access and retention in people living without HIV, especially in marginalized and minoritized populations [3, 7, 8].

Across the U.S., many clinics including the University of Alabama Birmingham (UAB) 1917 HIV Clinic Office-Based Opioid Treatment (OBOT) program began offering MOUD, specifically buprenorphine, via telehealth during this time. Preliminary data suggest that care retention in OBOT during this time was excellent [9], but there is little data to demonstrate the safety and effectiveness of these encounters relative to traditional in-person encounters or a hybrid approach (e.g. some combination of in-person and telehealth encounters). Specifically, it remains uncertain whether a dose-response relationship exists between telehealth exposure and important OUD and HIV care outcomes.

We hypothesized that OUD and HIV outcomes among PWH treated at the UAB OBOT Clinic would be similar between those with high telehealth utilization and those with less telehealth utilization during the COVID-19 public health emergency.

## Methods

We performed a retrospective cohort study of PWH seen at least once in the UAB 1917 HIV OBOT Clinic between 3/30/2020 and 4/30/2021. This period was selected as telehealth visits were encouraged (but not required) based on institutional and state policy [9]. The OBOT clinic is part of the UAB 1917 Clinic, a large, Ryan White-funded HIV clinic that provides HIV and primary care services to a panel of over 3500 patients to a heavily underserved and disproportionately rural population primarily in Central Alabama. While some allied health professional telehealth visits did occur during this time (peers, nurses), only provider visits were included in this study. The option of video or phone telehealth visit was offered to all patients; in cases where video visits were unable to be completed for technical reasons or access issues, phone visits were used instead.

Our primary outcome of interest was 6-month retention on buprenorphine with secondary outcomes of viral load suppression, mortality, and hospital admission. Of note, between 3/30/2020 and 7/31/2020, telehealth visits were very strongly encouraged due to a combination of a state “stay at home” order as well as a variety of more stringent institutional policies. The period after this from 8/1/2020 through 4/30/2021 was included to evaluate lower telehealth exposure and provide a comparator group for the “stringent restriction” period of 3/30/2020 through 7/31/2020. We then evaluated the number and type of OBOT visits (in person vs. telehealth) in the 6-month period following the index visit (i.e. visits from 3/30/2020 through 10/27/2021) and calculated the proportion of all visits that were telehealth as the primary measure of a given patient’s total telehealth exposure. For the purposes of the bivariate analysis, we categorized participants based on the amount of telehealth exposure. We found the distribution of telehealth visits was approximately normal though with a heavy right skew; therefore, we categorized telehealth exposure by relation to the median. “High proportion telehealth utilization,” was defined as at or above the median proportion of telehealth visits, while “low proportion telehealth utilization” was defined as below the median proportion (supplemental Table 1). Heretofore, we will refer to categories as “high proportion” and “low proportion” for brevity. Additionally, we categorized patients into those seen for their sentinel visit during stringent restrictions on in person visits (from 3/30/2020 through 7/31/2020) and those seen during more liberalized restrictions on in person visits (from 8/1/2020 through 4/30/2021) to account for reduced probability of telehealth exposure based on date of entry into the cohort.

Sociodemographic variables including age, race, gender, insurance status, and housing status at baseline visit were obtained from the medical record using a standardized data abstraction tool. Other variables including baseline and 6-month HIV viral loads, baseline- and 6-month CD4 counts, 6-month buprenorphine retention, 6-month hospital admission, and 6-month mortality were abstracted manually by research staff (WB and JE). Viral load (copies/mL) and CD4 cell count (cells/mm^3^) were defined as the laboratory value closest to the time (+/− 6 months). Identical baseline and follow-up values were not excluded from analysis (this occurred with 12 [28%] CD4 values and 2 [5%] viral loads). When no value was available within this period, the value was marked as missing. Six-month retention on buprenorphine was defined as having an active prescription for buprenorphine as evidenced by prescription records or provider notes including encounter buprenorphine prescriptions. Mortality was defined as report of death in electronic health record (EHR). Hospital admission was defined as evidence of readmission in the EHR.

We performed univariate and bivariate statistics comparing persons with telehealth exposure (high versus low) as well as 6-month buprenorphine retention status (retained versus non-retained). We analyzed descriptive statistics using frequencies and percentages for categorical data and summarized continuous data with means, standard deviations, and ranges. We did not impute missing data for any variables. We conducted parametric and nonparametric tests as indicated (supplemental Tables 1 and 2). We then performed multivariable logistic regression to predict 6-month buprenorphine retention based on telehealth exposure, controlling for covariates identified as potentially significant on bivariate analyses (baseline VL suppression, insurance status, housing, and age). We assessed collinearity between covariates using correlation coefficients. We also conducted a sensitivity analysis using date of entry into the cohort (rather than proportion of encounters that were telehealth) as the exposure of interest. Analyses were conducted using JMP version 17.0.

## Results

A total of 42 patients were included in the analysis. The average age was 42.9 years (range: 26.7–63.4); 21 (50%) were male, and 31 (74%) identified as white with the other 11 (26%) identifying as black. Most (*n* = 26; 62%) were privately insured, while the others were either publicly insured (*n* = 14; 33%) or uninsured (*n* = 2; 5%). Most were permanently housed (*n* = 33; 79%). In terms of HIV control, most (*n* = 33; 79%) were virally suppressed at baseline, and the average baseline CD4 was 659 (range: 172–1340). There were 9 (21%) missing baseline CD4 values, 12 (29%) missing 6-month CD4 values, and 1 (2%) missing baseline and 6-month viral load values. There were a total of 247 completed encounters over the study period, of which 140 (57%) were in person, 61 (25%) were telephone visits, and 46 (19%) were televideo visits. A total of 17 (40.5%) patients had low telehealth utilization, while 25 (60%) had high utilization.

About half (*n* = 22; 52%) of the participants had their sentinel visit during the period of stringent telehealth policies, while 20 (48%) had their sentinel visit in latter period (supplemental Table 1). In terms of 6-month outcomes, 31 patients (74%) were retained on buprenorphine, 4 (10%) were admitted to the hospital, 0 died, and 35 (85%) were virally suppressed (6% more than baseline; McNemar’s Test with baseline viral suppression: *p* = 0.16).

Unadjusted bivariate analyses (supplemental Table 1) indicated that factors associated with a high telehealth utilization were non-private insurance status (*p* = 0.005) and sentinel visit during stringent telehealth restrictions (p = < 0.001). Viral load suppression at baseline visit was significantly associated with 6-month buprenorphine retention (*p* = 0.01), while private insurance status (*p* = 0.17), non-permanent housing status (*p* = 0.40), and lower age (*p* = 0.38), while not statistically significant, were relatively more predictive of six-month retention on buprenorphine than other variables (supplemental Table 2). A multivariable logistic regression model was constructed using these identified variables as covariates with proportion of telehealth visits as the main independent variable of interest.

A test of the full model compared with a constant-only or null model was statistically significant (χ2(5) = 11.61; *p* = 0.04), indicating good model fit. There was no evidence of multicollinearity as the highest correlation coefficient was 0.52. The full model (see Table 1) demonstrated that only baseline viral load suppression (*p* = 0.008) was significantly associated with six-month buprenorphine retention. An increased proportion of telehealth appointments (continuous variable) was non-statistically significantly associated with higher odds of 6-month buprenorphine retention (aOR = 1.87, 99% CI 0.07–51.15). Sensitivity analysis using the alternative case definition of date of entry into the cohort (rather than proportion of encounters that were telehealth) as the exposure of interest demonstrated identical prediction results (supplemental Table 3).

Regarding secondary outcomes, unadjusted bivariate analyses indicated that 6-month viral load was predicted by baseline viral load (McNemar’s Test *p* = 0.16), so no multivariable analysis was performed. No multivariable analysis was performed for the outcome of 6-month admission as no model performing better than null could be identified. No patients died during follow-up.

## Discussion

In this study of outpatient PWH with OUD in care at a Ryan White HIV/AIDS Program-funded HIV clinic in the Deep South, we found that a higher proportion of telehealth use was associated with similar retention on buprenorphine, viral load suppression, and hospitalization, consistent with our hypothesis. Importantly from an equity standpoint, race, older age, and lack of housing did not significantly impact telehealth utilization. Similarly, PWH who were uninsured and with public insurance had similar rates of telehealth utilization as privately insured peers. These findings highlight that telehealth was accessible to our most vulnerable PWH with OUD, many of whom are at greatest risk of HIV viremia and transmission (i.e., the unhoused and uninsured). Furthermore, the ability of older PWH to access telehealth at similar rates to younger groups suggests that telehealth for OUD care does not exacerbate the digital divide. Together, these findings point to telehealth as a safe means to improve health equity for the most marginalized PWH: those living with addiction in a poor and rural state.

Only baseline viral suppression status was a significant predictor of retention in multivariate modeling, reflecting the known relationship between MOUD use and virologic response in PWH with comorbid OUD [10]. The overall rate of 6-month retention in care we report here (74%) is better than those reported in other studies in the general population with OUD [4, 7]. This likely reflects the benefit of integrating OUD care into Ryan White HIV/AIDS-funded medical homes as well as the relative stability and high care engagement of our outpatient population of PWH [10]. Similar to other studies, we found a high rate of telephone utilization (25% of all visits), indicating that the low barrier of telephone use (audio only) was likely important in promoting care access [7, 11]. Given race and geography-related disparities in access to digital technologies, efforts should be made to preserve this important route of access to telehealth services [11, 12].

This study was limited by its single-center nature and relatively low number of participants. Additionally, measures of CD4 count were limited by a high number of missing data. This limited the types of analyses that could be meaningfully performed with the data. Finally, the UAB 1917 OBOT clinic is a very well-supported clinic with extensive access to such wrap-around services as transportation assistance and copay assistance. Whether these results would be applicable to a clinic with fewer resources is not clear, and this question represents a potential focus for future studies.

Given the ongoing substantial contribution of injection drug use and related behaviors (e.g. exchange of sex for drugs or money) to the HIV epidemic, we must ensure that barriers remain low to access critical, life-saving OUD treatment for all, but most of all for PWH. While retrospective and limited in size, this study suggests that such alternate care models as telehealth-based hub-and-spoke care delivery may be safe and effective while enhancing health equity in people living with HIV and OUD. However, with the lifting of pandemic-era regulations, restrictions on the use of telehealth by payers and governments have returned [5]. While there remains much work to be done to establish the optimal role for telehealth in treatment of substance use disorders, access to this important intervention is clearly accessible and safe for many patients, including PWH. To reduce HIV transmission and meet the bold and ambitious goals of the EHE initiative, telehealth access must be maintained.

## Supplementary Material

Supplemental Material 1

## Figures and Tables

**Table 1:** Multivariable-adjusted associations of covariates with 6-month buprenorphine retention

Variable	aOR	*p*-value	aOR 95% CI
Baseline viral load suppression (reference = suppressed)	15.28	0.017	1.63–143.36
Insurance (reference = non-private)	0.64	0.69	0.07–5.67
Proportion of telehealth	1.87	0.71	0.07–51.15
Housing (reference = permanent)	0.24	0.31	0.02–3.67
Age	0.93	0.22	0.82–1.05

Abbreviations: aOR, adjusted odds ratio; CI, confidence interval

## Data Availability

The datasets used and/or analyzed during the current study are available from the corresponding author on reasonable request.

## References

[1] Fauci AS, Redfield RR, Sigounas G, Weahkee MD, Giroir BP. Ending the HIV Epidemic: a plan for the United States. Jama Mar. 2019;5(9):844–5. 10.1001/jama.2019.1343 30730529

[2] Gagnon KW, Bradford W, Bassler J, Community-based Services for Hospitalized Patients with serious injection-related infections in Alabama: a brief report. Open Forum Infectious Diseases; 2024. 10.1093/ofid/ofae231 PMC11134460 38813257

[3] Mahmoud H, Naal H, Whaibeh E, Smith A. Telehealth-based delivery of medication-assisted treatment for opioid use disorder: a critical review of recent developments. Curr Psychiatry Rep Sep. 2022;24(9):375–86. 35895282 10.1007/s11920-022-01346-z PMC9326140

[4] Weintraub E, Greenblatt AD, Chang J, Himelhoch S, Welsh C. Expanding access to buprenorphine treatment in rural areas with the use of telemedicine. Am J Addict Dec. 2018;27(8):612–7. 30265425 10.1111/ajad.12805

[5] Glenn JH Sen. Robert’s telehealth bill passes Senate. Alabama Political Reporter. March 30, 2022, 2022.

[6] Walters SM, Perlman DC, Guarino H, Mateu-Gelabert P, Frank D. Lessons from the First Wave of COVID-19 for Improved medications for Opioid Use Disorder (MOUD) treatment: benefits of Easier Access, Extended take homes, and New Delivery modalities. Subst Use Misuse. 2022;57(7):1144–53. 35443862 10.1080/10826084.2022.2064509 PMC9709780

[7] Frost MC, Zhang L, Kim HM, Lin LA. Use of and Retention on Video, Telephone, and In-Person Buprenorphine Treatment for Opioid Use Disorder during the COVID-19 pandemic. JAMA Netw Open Oct. 2022;3(10):e2236298. 10.1001/jamanetworkopen.2022.36298 PMC9557869 36223118

[8] Hailu R, Mehrotra A, Huskamp HA, Busch AB, Barnett ML. Telemedicine Use and Quality of Opioid Use Disorder Treatment in the US during the COVID-19 pandemic. JAMA Netw Open Jan. 2023;3(1):e2252381. 10.1001/jamanetworkopen.2022.52381 PMC10038015 36692880

[9] Eaton EF, Tamhane A, Turner W, Raper JL, Saag MS, Cropsey KL. Safer in care: a pandemic-tested model of integrated HIV/OUD care. Drug Alcohol Depend Feb. 2022;1:231:109241. 35007957 10.1016/j.drugalcdep.2021.109241 PMC9758363

[10] McNamara KF, Biondi BE, Hernández-Ramírez RU, Taweh N, Grimshaw AA, Springer SA. A systematic review and Meta-analysis of studies evaluating the Effect of Medication Treatment for Opioid Use Disorder on Infectious Disease outcomes. Open Forum Infect Dis Aug. 2021;8(8):ofab289. 34430670 10.1093/ofid/ofab289 PMC8378589

[11] Lin LA, Zhang L, Kim HM, Frost MC. Impact of COVID-19 Telehealth Policy Changes on Buprenorphine Treatment for Opioid Use Disorder. Am J Psychiatry Oct. 2022;179(10):740–7. 35899380 10.1176/appi.ajp.21111141 PMC9529783

[12] Thomas-Jacques T, Jamieson T, Shaw J. Telephone, video, equity and access in virtual care. NPJ Digit Med Nov. 2021;18(1):159. 10.1038/s41746-021-00528-y PMC8602296 34795356

