# Supplementary material for: Equal Access, Equal Outcomes: Telehealth Utilization Around the COVID-19 Pandemic among People Living with HIV and Opioid Use Disorder in the Deep South
Source: AIDS Behav. 2024 Nov 15;29(2):684–8. doi: 10.1007/s10461-024-04550-5 (PMC11813669; doi:10.1007/s10461-024-04550-5)
Supplement: Supplementary file 1 — Supplementary Material 1 [file 10461_2024_4550_MOESM1_ESM.docx]

Supplemental Tables

**Table 1.** Univariate and bivariate analyses by telehealth exposure status.

|  | **Low Proportion telehealth utilization (n=17)** | **High proportion telehealth utilization (n=25)** | **Total (N=42)** | ***p*-value** |
| --- | --- | --- | --- | --- |
| **Age** |  |  |  | 0.60^1^ |
| Mean (SD) | 41.9 (8.6) | 43.5 (9.9) | 42.9 (9.4) |  |
| Range | 31.1 - 58.4 | 26.7 - 63.4 | 26.7 - 63.4 |  |
| **Gender** |  |  |  | 0.75^2^ |
| Male | 9 (52.9%) | 12 (48.0%) | 21 (50.0%) |  |
| Non-male | 8 (47.1%) | 13 (52.0%) | 21 (50.0%) |  |
| **Race** |  |  |  | 0.70^2^ |
| White | 12 (70.6%) | 19 (76.0%) | 31 (73.8%) |  |
| Black | 5 (29.4%) | 6 (24.0%) | 11 (26.2%) |  |
| **Insurance** |  |  |  | **0.005^3^** |
| Private | 15 (88.2%) | 11 (44.0%) | 26 (61.9%) |  |
| Non-private | 2 (11.8%) | 14 (56.0%) | 16 (38.1%) |  |
| **Housing** |  |  |  | 1.00^3^ |
| Permanent | 13 (76.5%) | 20 (80.0%) | 33 (78.6%) |  |
| Non-permanent | 4 (23.5%) | 5 (20.0%) | 9 (21.4%) |  |
| **Baseline CD4** |  |  |  | 0.87^1^ |
| Mean (SD) | 647.8 (219.8) | 665.6 (386.7) | 658.6 (327.1) |  |
| Range | 301 - 1094 | 172 - 1340 | 172 - 1340 |  |
| **Baseline VL suppression** |  |  |  | 1.00^3^ |
| Suppressed | 14 (82.4%) | 19 (76.0%) | 33 (78.6%) |  |
| Non-suppressed | 3 (17.6%) | 5 (20.0%) | 8 (19.0%) |  |
| **Entry into cohort** |  |  |  | **<0.001**^3^ |
| Prior to 7/31/20 | 2 (11.8%) | 20 (80.0%) | 22 (52.4%) |  |
| After 7/31/20 | 15 (88.2%) | 5 (20.0%) | 20 (47.6%) |  |
| **Phone visits** |  |  |  | NP |
| Mean (SD) | 0.4 (0.6) | 2.2 (1.7) | 1.5 (1.6) |  |
| Range | 0 - 2 | 0 - 6 | 0 - 6 |  |
| **Televideo visits** |  |  |  | NP |
| Mean (SD) | 1.6 (1.9) | 2.2 (1.7) | 1.1 (1.6) |  |
| Range | 0 - 7 | 0 - 6 | 0 - 7 |  |
| **In person visits** |  |  |  | NP |
| Mean (SD) | 4.6 (1.8) | 2.4 (1.6) | 3.3 (2.0) |  |
| Range | 1 - 7 | 0 - 6 | 0 - 7 |  |
| **No show visits** |  |  |  | NP |
| Mean (SD) | 0.6 (1.0) | 1.1 (1.2) | 0.9 (1.1) |  |
| Range | 0 - 4 | 0 - 3 | 0 - 4 |  |
| **Six-month buprenorphine retention** |  |  |  | 0.73^3^ |
| Retained | 5 (29.4%) | 19 (76.0%) | 31 (73.8%) |  |
| Not retained | 12 (70.6%) | 6 (24.0%) | 11 (26.2%) |  |
| **Six-month admission** |  |  |  | 0.64^3^ |
| Admitted | 1 (5.9%) | 3 (12.0%) | 4 (9.5%) |  |
| Not admitted | 16 (94.1%) | 22 (88.0%) | 38 (90.5%) |  |
| **Six-month mortality** |  |  |  | NP |
| Died | 0 (0.0%) | 0 (0.0%) | 0 (0.0%) |  |
| Did not die | 17 (100.0%) | 25 (100.0%) | 42 (100.0%) |  |
| **Six-month CD4 count** |  |  |  | 0.83^1^ |
| Mean (SD) | 626.3 (215.2) | 647.5 (317.8) | 639.0 (277.3) |  |
| Range | 365 - 1083 | 273 - 1284 | 273 - 1284 |  |
| **Six-month VL suppression** |  |  |  | 1.00^3^ |
| Suppressed | 15 (88.2%) | 20 (83.3%) | 35 (85.4%) |  |
| Not suppressed | 2 (11.8%) | 4 (16.7%) | 6 (14.6%) |  |

^*^ *High telehealth utilization” refers to proportion of telehealth visits at or above the median, while “low telehealth utilization” refers to proportion of telehealth visits below the median.*

^1^Independent t-test

^2^Pearson chi-square test

^3^Fisher’s exact test (two-tail)

Abbreviations: NP, hypothesis testing not performed; SD, standard deviation; VL, viral load

**Table 2.** Univariate and bivariate analyses by 6-month buprenorphine retention.

|  | **Retained (n=31)** | **Not retained (n=11)** | **Total (N=42)** | ***p-*value** |
| --- | --- | --- | --- | --- |
| **Age** |  |  |  | 0.38^1^ |
| Mean (SD) | 42.1 (9.2) | 45.1 (10.0) | 42.9 (9.4) |  |
| Range | 26.7 - 58.4 | 30.4 - 63.4 | 26.7 - 63.4 |  |
| **Gender** |  |  |  | 1.00^3^ |
| Male | 16 (51.6%) | 5 (45.5%) | 21 (50.0%) |  |
| Non-male | 15 (48.4%) | 6 (54.5%) | 21 (50.0%) |  |
| **Race** |  |  |  | 0.73^3^ |
| White | 24 (77.4%) | 7 (63.6%) | 31 (73.8%) |  |
| Black | 7 (22.6%) | 4 (36.4%) | 11 (26.2%) |  |
| **Insurance** |  |  |  | 0.17^3^ |
| Private | 22 (71.0%) | 4 (36.4%) | 26 (61.9%) |  |
| Non-private | 9 (29.0%) | 7 (63.6%) | 16 (38.1%) |  |
| **Housing** |  |  |  | 0.40^3^ |
| Permanent | 23 (74.2%) | 10 (90.9%) | 33 (78.6%) |  |
| Non-permanent | 8 (25.8%) | 1 (9.1%) | 9 (21.4%) |  |
| **Baseline CD4** |  |  |  | 0.86^1^ |
| Mean (SD) | 664.4 (348.5) | 640.3 (268.7) | 658.6 (327.1) |  |
| Range | 172 - 1340 | 273 - 1094 | 172 - 1340 |  |
| **Baseline VL suppression** |  |  |  | **0.01**^3^ |
| Suppressed | 28 (90.3%) | 5 (45.5%) | 33 (78.6%) |  |
| Non-suppressed | 3 (9.7%) | 5 (45.5%) | 8 (19.0%) |  |
| Missing | 0 (0.0%) | 1 (9.1%) | 1 (2.4%) |  |
| **Entry into cohort** |  |  |  | 0.59^2^ |
| Prior to 7/31/20 | 17 (54.8%) | 5 (45.5%) | 22 (52.4%) |  |
| After 7/31/20 | 14 (45.2%) | 6 (54.5%) | 20 (47.6%) |  |
| **Phone visits** |  |  |  | 0.20^4^ |
| Mean (SD) | 1.7 (1.7) | 0.9 (1.2) | 1.5 (1.60) |  |
| Range | 0 - 6 | 0 - 3 | 0 - 6 |  |
| **Televideo visits** |  |  |  | 0.85^4^ |
| Mean (SD) | 1.2 (1.8) | 0.9 (1.0) | 1.1 (1.6) |  |
| Range | 0 - 7 | 0 - 3 | 0 - 7 |  |
| **In person visits** |  |  |  | 0.08^4^ |
| Mean (SD) | 3.6 (1.7) | 2.5 (2.5) | 3.3 (2.0) |  |
| Range | 0 - 6 | 0 - 7 | 0 - 7 |  |
| **No show visits** |  |  |  | **0.001**^4^ |
| Mean (SD) | 0.6 (0.9) | 1.8 (1.1) | 0.9 (1.1) |  |
| Range | 0 - 3 | 0 - 4 | 0 - 4 |  |
| **Telehealth proportion** |  |  |  | 0.46^1^ |
| Mean (SD) | 0.39 (0.29) | 0.48 (0.38) | 0.42 (0.31) |  |
| Range | 0 - 1 | 0 - 1 | 0 - 1 |  |
| **Six-month admission** |  |  |  | 0.28^3^ |
| Admitted | 2 (6.5%) | 2 (18.2%) | 4 (9.5%) |  |
| Not admitted | 29 (93.5%) | 9 (81.8%) | 38 (90.5%) |  |
| **Six-month mortality** |  |  |  | NP |
| Died | 0 (0.0%) | 0 | 0 (0.0%) |  |
| Did not die | 31 (100.0%) | 11 (100.0%) | 42 (100.0%) |  |
| **Six-month CD4 count** |  |  |  | 0.36^1^ |
| Mean (SD) | 649.5 (289.1) | 610.3 (257.9) | 639.0 (277.3) |  |
| Range | 277 - 1284 | 273 - 984 | 273 - 1284 |  |
| **Six-month VL suppression** |  |  |  | **0.02**^3^ |
| Suppressed | 29 (93.5%) | 6 (54.5%) | 35 (83.3%) |  |
| Not suppressed | 2 (6.5%) | 4 (36.4%) | 6 (14.3%) |  |
| Missing | 0 (0.0%) | 1 (9.1%) | 1 (2.4%) |  |

^1^Independent t-test

^2^Pearson chi-square test

^3^Fisher’s exact test

^4^Mann-Whitney U Test

Abbreviations: NP, hypothesis testing not performed; SD, standard deviation; VL, viral load

**Table 3.** Sensitivity analysis using date of cohort entry in lieu of proportion of telehealth encounters as the exposure.

| Variable | aOR | *p*-value | aOR 95% CI |
| --- | --- | --- | --- |
| Baseline viral load suppression (reference = suppressed) | 14.67 | **0.019** | 1.57 - 137.27 |
| Insurance (reference = non-private) | 0.60 | 0.63 | 0.08 - 4.85 |
| Entry into Cohort (reference = before) | 1.87 | 0.52 | 0.28 - 12.52 |
| Housing (reference = permanent) | 0.23 | 0.28 | 0.02 - 3.34 |
| Age | 0.93 | 0.24 | 0.82 - 1.05 |

Abbreviations: aOR, adjusted odds ratio; CI, confidence interval
